# Similar representation of names and faces in the network for person perception

**DOI:** 10.1016/j.neuroimage.2023.120100

**Published:** 2023-07-01

**Authors:** Aidas Aglinskas, Scott L. Fairhall

**Affiliations:** a Department of Psychology and Neuroscience, Boston College, Chestnut Hill, MA 02467, USA; b Center for Mind/Brain Sciences (CIMeC), University of Trento, Rovereto, TN 38068, Italy

**Keywords:** Person knowledge, fMRI, Cortical network, Face perception, Representational similarity analysis

## Abstract

Person-knowledge encompasses the diverse types of knowledge we have about other people. This knowledge spans the social, physical, episodic, semantic & nominal information we possess about others and is served by a distributed cortical network including core (perceptual) and extended (non-perceptual) subsystems. Our understanding of this cortical system is tightly linked to the perception of faces and the extent to which cortical knowledge-access processes are independent of perception is unclear. In this study, participants were presented with the written names of famous people and performed ten different semantic access tasks drawn from five cognitive domains (biographic, episodic, nominal, social and physical). We used representational similarity analysis, adapted to investigate network-level representations (NetRSA) to characterise the inter-regional functional coordination within the non-perceptual extended subsystem across access to varied forms of person-knowledge. Results indicate a hierarchical cognitive taxonomy consistent with that seen during face-processing and forming the same three macro-domains: socio-perceptual judgements, episodic-semantic memory and nominal knowledge. The coordination across regions was largely preserved within elements of the extended system associated with internalised cognition but differed in prefrontal regions. Results suggest the elements of the extended system work together in a consistent way to access knowledge when viewing faces and names but that coordination patterns also change as a function of input-processing demands.

## Highlights

• Cortical representation about conspecifics is largely stable regardless of stimulus modality (viewing faces vs reading names).
• Diverse kinds of knowledge about other people falls into three macro categories of cortical signatures: memories (episodic and biographical knowledge), traits (physical and social knowledge) and knowledge of names.
• Some regions involved in person-knowledge (IFG and OFC) change their functional coordination patterns within the network, while others (regions involved in internalized cognition) maintain stable functional coupling.

## 1 Introduction

To efficiently interact with conspecifics, we need to not only recognise the identity of the individual, but also to access the diverse kinds of information we associate with them (memories, facts, social information, names). Such person-related information about a familiar individual can be accessed both when we see their face and when we read their name. However, the extent to which the neural mechanisms underlying this process differ between face-viewing and name-reading remain uncertain.

Originally conceptualised as a face-processing system, the network of brain regions thought to underlie perceiving and knowing about others has been extensively studied (Fairhall and Ishai, 2007; Gobbini and Haxby, 2007; Haxby et al., 2001; Haxby et al., 2000). This distributed cortical network has been divided into core and extended systems (Haxby et al., 2000). The ”core system” consists of occipital and fusiform face areas (OFA, FFA; Gauthier et al., 2000; Kanwisher et al., 1997) and posterior superior temporal sulcus (pSTS) and is primarily involved in perceptual processing. The extended system is activated more when viewing familiar than unfamiliar faces and consists of regions associated with non-perceptual person-related cognition. These regions include the inferior frontal gyrus (IFG), orbitofrontal cortex (OFC), amygdala, ventromedial and dorsomedial prefrontal cortices (vmPFC, dmpFC), anterior temporal lobe (ATL), angular gyrus (AG), precuneus, and potentially—the recently identified, face-selective anterior temporal face patch (ATFP; Gobbini and Haxby, 2007; Rajimehr et al., 2009; Tsao et al., 2008; Von Der Heide et al., 2013).

The role of the extended system in access to information about others in the absence of faces is less well understood. When viewing famous faces, the fMRI recruitment of these regions requires longer time spans compared to recruitment of core components (Ubaldi and Fairhall, 2021b). While elements of the core system are not selectively activated by person-related name stimuli (Bi et al., 2016), some regions of the extended system do exhibit a preference for person-related word stimuli. The names of famous people selectively activate components of the extended system compared to common-nouns referencing face parts (Wang et al., 2016) and names of famous places (Fairhall et al., 2014) and are also selectively activated when participants access information about general kinds of people (e.g. “professions”; Fairhall and Caramazza 2013), when processing more complex, sentence-level, stimuli involving people (Rabini et al., 2021) or when retrieving stored encyclopaedic knowledge relating to people (Ubaldi et al., 2022). These findings indicate at least some commonality in the representation of person-knowledge for face- and name-cued cognition*.* However, it remains uncertain how name and face stimuli alter the access to person-related information in the extended system and whether these differences (if they exist) would be reflected in regional changes, or more diffuse differences in regional coordination.

In a recent fMRI study we showed that, when viewing faces and accessing varied forms of information about the pictured individual, the diverse kinds of person-knowledge are enabled by the coordinated interactions between regions (Aglinskas and Fairhall, 2020). Specifically, for a set of 40 famous faces, we had participants retrieve information about these famous individuals from one of five knowledge domains - nominal, episodic, biographical, social and physical - at different times in the experiment. We observed that activity in different components of the network for perceiving and knowing about others showed relative rather than absolute variations in regional preferences for different knowledge domains. Subtle and reliable differences were evident across the network when different knowledge domains were accessed, rather than network components being switched on or off during access to different knowledge variants. These variations were utilised in a multivariate analysis (NetRSA) that exploited regional and cognitive profiles across nodes of the face-processing system.

NetRSA leverages the same principles as traditional voxel-level RSA. With voxel level RSA, more similar regional neural-patterns produced by stimuli can be used to infer more similar processing of those stimuli and reconstruct a space which indicates how different kinds of stimuli are represented within a brain region. netRSA can be used in the same way but applied to a network, and in our case different cognitive domains. We refer to this as the cognitive taxonomy of person-knowledge. At the same time, voxel-level RSA could also be used to look at the similarity of the response profiles of different voxels across a range of stimuli and group these voxels into functional clusters. This would be challenging to interpret at the voxel level, however when applied at the level of the network this approach allows us to gain insight into the functional organisation of the network by looking at how closely brain regions work together to accomplish the varied forms of knowledge representation about people. This in turn allows us to determine neural coordination of elements of the person-selective network, which we term regional taxonomy in the present work.

In our previous experiment, this approach revealed a hierarchical cognitive taxonomy based on the cognitive tuning profiles of different network nodes, which grouped physical with social knowledge, biographic with episodic knowledge, and separately, nominal knowledge when viewing famous faces. At the same time, comparing regional coordination profiles across varied forms of information access, we were able to decompose the network into three subcomponents that reliably work together to accomplish cognitive goals: a perceptual-prefrontal subsystem (core regions, OFC, IFG), an intrinsic/default mode network (DMN) subsystem (precuneus, vmPFC, lateral ATL, angular gyrus) and an anterior ventral subsystems (ATFP, amygdala). In this way, the NetRSA approach allowed us to characterise both the relationship between different forms of person-knowledge and the coordination between different brain regions across varied forms of cognitive access.

In the present study, we leverage this approach to investigate whether the cortical implementation of access to person-knowledge in the extended system is reshaped when knowledge is accessed *via* words instead of faces. Specifically, the use of name stimuli instead of face stimuli allows us to confirm that the effects attributed to knowledge access indeed relate to this processes and not to differences in perceptual processes associated with the ten different tasks while at the same time enforcing a semantic route in processing - breaking apart direct emotional responses to faces or automatic processing related to physical characteristics such as attractiveness or even trustworthiness (Oosterhof and Todorov, 2008). Finally, in addition to showing the generalisation of previously reported effects, this study provides replication and generalisation of previous findings. As in the previous study, while reading names of famous people, participants recalled ten different types of knowledge spanning five domains of person-knowledge (social, physical, episodic, biographical & nominal). Through NetRSA, we exploit the rich information present in the regional response profile across access to varied forms of person-related information to characterise the relationships between these processes. We predict that if cognitive processes are consistent across modalities, we will observe a preserved relationship between different tasks/cognitive domains across modalities while alterations in this organisation will indicate reorganisation across modalities.

## 2 Methods

### 2.1 Participants

Thirty-one participants took part in this study (mean age M = 24.74, SD = 3.13). All participants had normal or corrected to normal vision and no history of neurological disorders. Procedures were approved by the University of Trento Ethical committee. Participants gave written informed consent and were compensated for their time. Seven participants were excluded prior to analysis. Two of these participants did not complete the experiment due to technical issues with the stimulation computer and data from five participants were excluded due to excessive head-motion in the scanner. The final subject pool consisted of 24 subjects.

In the present study, the analyses central for inference are the netRSA analysis comparing the observed representational space to the theoretical models (see bar plots of Figs. 4 and 5). To estimate the expected effects size, we considered comparable netRSA analysis from our previous work and found that effect size estimates ranged from 1.4 to 1.9 (Cohen's D). With the current sample of 24 we can expect to detect an effect size as low as 0.53 with a beta of 0.8, indicating that the present study can be considered relatively well-powered.

### 2.2 Stimuli

Stimuli were 40 names of recognisable famous people (politicians, actors, athletes or business people). First and last names were presented in large font on separate lines. Mean name length was M = 12.15, SD = 2.59 letters. For monuments control task, stimuli were written names of familiar places presented in Italian ("Notre Dame”, “Piramidi di Ghiza”, etc.). Person names and monument names did not differ in overall length (t(78) = 0.30,p = .768, independent sample t-test).

### 2.3 Task

**Fig. 1:**
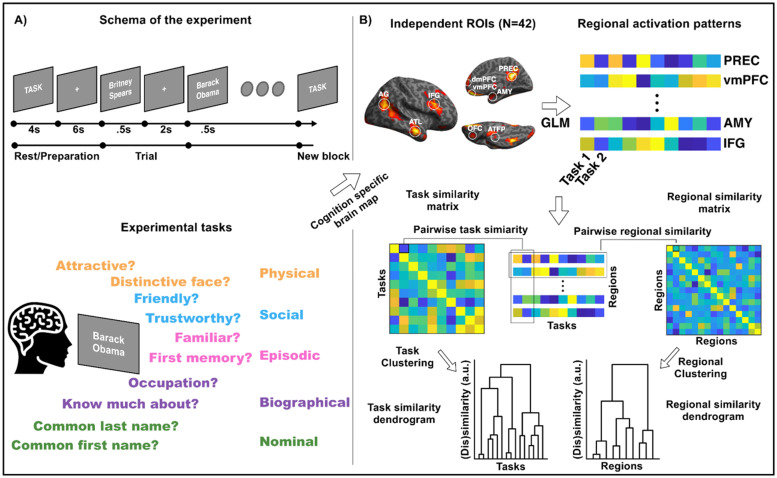
Experimental and data analysis procedures. (A) Schematic of the fMRI experiment: Each block consisted of 8 stimuli presented for 0.5 s each, followed by 2 s of fixation cross during which participants provided responses. Experimental tasks: Participants recalled ten different types of knowledge sampled from 5 domains of person knowledge. In addition to experimental tasks, there were two 1-back matching control tasks using either names of famous people (same people used for experimental tasks) or famous monuments. **(**B) Analysis schematic: task specific response estimates (beta values) for each task were extracted from independently defined, face-selective ROIs. This resulted in a 10 value vector (each value representing response magnitude for each person-knowledge task) for each ROI. Response vectors were then correlated to obtain task-similarity and ROI-similarity matrices. ROI similarity matrices were entered into a clustering analysis to find groups of regions similarly responding across tasks. Task similarity matrices were entered into a clustering analysis to find groups of tasks sharing common neural signatures.

**Fig. 2:**
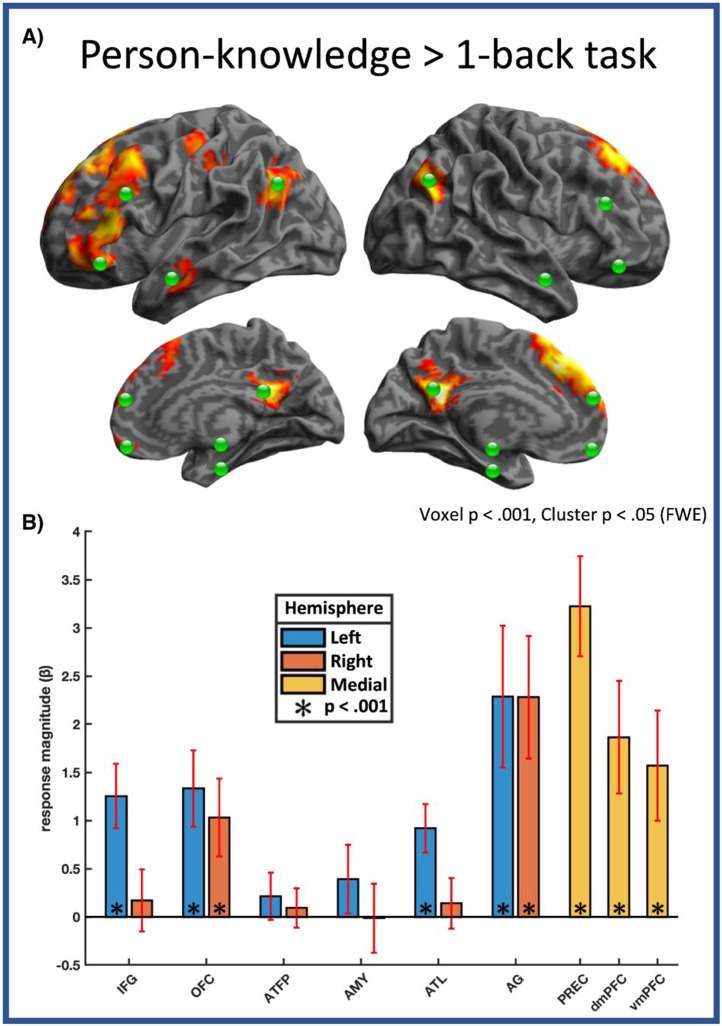
Name-cued access to person-knowledge**. (**A) Whole brain map showing regions more active during person-knowledge tasks than 1-back famous person name matching task. Regions associated with internalised cognition (precuneus, vmPFC, dmPFC, left ATL, bilateral AG, left OFC) are activated. Responses in other key regions active during face-cued person-knowledge retrieval (amygdala, ATFP, right OFC, right ATL) were not significant after cluster correction. Green dots indicate the loci of ROIs derived from an independent set of participants**. (**B) Independent ROI analysis of face-selective extended system regions’ involvement in access to person-knowledge during name reading.

**Fig. 3:**
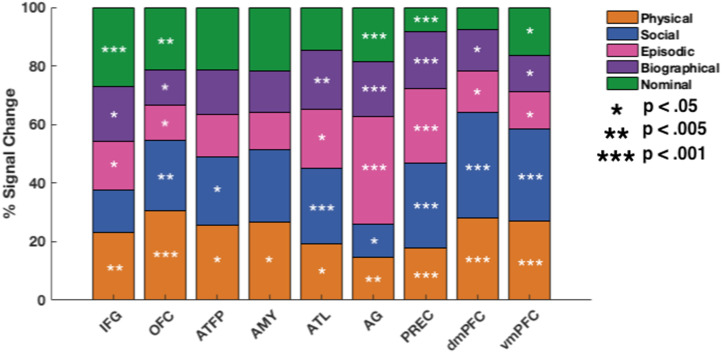
Regional preference patterns. Percentage of total activation elicited by each cognitive domain. Stars denote significance of regional activation for each task, compared to the name-matching baseline condition (task > name matching 1-back, uncorrected). Most regions are involved in most cognitive domains, and no brain region is driven by a single cognitive domain. However subtle patterns can be seen in the variations of loadings of particular domains in different brain regions.

Each experimental block started with a 4 s instruction screen specifying the task, followed by a 6 s fixation cross. After that a name was presented for 0.5 s followed by 2 s of fixation cross during which subjects provided a response *via* a button box. Within each 8-trial block, participants were instructed to respond to a question from one of the five knowledge domains of person-knowledge: episodic memories, semantic knowledge, social judgments, nominal knowledge and physical knowledge. For each category, we chose two different probe questions that required access to each kind of knowledge (totalling ten experimental tasks; see Fig. 1 and Table 1). The five knowledge-domains were selected based on a review of the relevant neuromage literature and were formulated to incorporate the cognitive process most commonly used to explain activation of the various elements of the network for perceiving and knowing about others. A subset of the ten tasks were selected based on previous use in the literature (e.g. distinctiveness, attractiveness, familiarity, occupation, trustworthiness and friendliness (e.g. Kawamura and Komori, 2008; Pandeirada et al., 2020; Bohrn et al., 2013; Fairhall and Caramazza, 2013; Bzdok et al., 2011). For others we designed them based on congruency with the desired cognitive domain (how many facts, first memory, name-knowing confidence and commonness of name). These experimental tasks were the same as those in Aglinskas and Fairhall (2020) with one modification. The Nominal Task used in the face experiment, “How well can you recall this person's full name?” was changed to “How common is this person's last name” for the present experiment. These tasks were informally piloted on about 7 individuals to check that tasks were easily performed and participants felt (during post-session debriefing) that they fit into the relevant cognitive domain. We had some confidence in the result of this approach based on the results of Aglinskas and Fairhall (2020), where tasks were seen to match to knowledge-domain partners in their representation across the cortex (Figs. 2 and 3).

**Table 1:** Task description. For each of the five person-knowledge domains investigated, we choose two representative probe questions, totalling ten experimental conditions.

Knowledge Category	Task Cue	Participant Instructions	Answer Choices
Nominal	Common name	How common is this person's first name?	Likert scale (1–4)
	Common surname	How common is this person's last name?	Likert scale (1–4)
Physical	Attractive	How attractive do you find this person?	Likert scale (1–4)
	Distinctive	How distinctive is this person's face?	Likert scale (1–4)
Social	Friendly	How friendly is this person?	Likert scale (1–4)
	Trustworthy	How trustworthy is this person?	Likert scale (1–4)
Episodic	Familiar	How familiar is this person to you?	Likert scale (1–4)
	First memory	For how long have you known this person?	Likert scale (1–4)
Biographical	How many facts	How many facts can you recall about this person?	Likert scale (1–4)
	Occupation	What is this person's occupation?	Predefined categories (see “task”)

In addition, there were two 1-back matching control tasks that utilised either the names of people or of famous monuments. Both control tasks required the participant to press a button indicating if the presented stimulus was the same as or different to the preceding trial. The experiment consisted of five runs (8 min, 42 s each). Sixteen blocks were presented in a randomised order (one block for each task plus three repetitions each of face and monument 1-back control blocks). Participants responded *via* a button box. For 9/10 tasks the rating scale was a 1–4 Likert scale. For the occupation question (“what is this persons’ occupation”), answer choices had predefined categories (1 = actor or TV presenter, 2 = singer or musician, 3 = politician or sportsman, 4 = other or none of the above). Prior to scanning, participants practised answering experimental questions on a different set of famous people repeating each question for five trials.

### 2.4 Data acquisition

Participants were scanned at the center for Mind/Brain Sciences (CIMeC), University of Trento, Italy. Data was collected using Bruker BioSpin MedSpec 4T, with 8-channel phased-array head coil. Five runs of 209 echo-planar volumes, consisting of 34, AC-PC aligned axial slices were acquired while participants performed the task (FOV = 64 mm x 64 mm, TR = 2.5 s, TE = 33, FA = 73°). Voxel size was 3 × 3 × 3 mm with a 1 mm gap. In addition to functional data, a whole brain T1 MPRAGE anatomical image was acquired (whole brain (FOV = 256×224, 176 1 mm axial slices).

### 2.5 Data analysis

Data were pre-processed with SPM12. Functional images were realigned to account for motion, grey matter segmented, warped into MNI space and smoothed with an 8 mm FWHM kernel. Subject specific response estimates (beta weights) were derived by fitting a general linear model (GLM) to the data. 12 regressors (ten experimental tasks, two control tasks) were included as explanatory variables. Six motion parameters from the re-alignment procedure were included as regressors of no interest.

### 2.6 Regions of interest

Regions of interest (ROIs) were selected from an independent (N=42) experiment, using a separate pool of subjects. While this did not allow us to locate individual subject-specific ROIs it did enable comparisons of cognitive response in face-selective regions across the two studies (current experiment and face experiment) without bias to either dataset. In the localiser experiment, a separate group of participants performed a 1-back matching task with 12 s blocks of famous faces, common animals or common objects. ROIs were identified across the whole brain in a data-driven way. The contrast faces > animals + common objects (P < .05 family-wise error (FWE) corrected) was used to identify face-selective peaks more than 30 mm apart. A total of 21 peaks were identified that converged with previously reported face and person selected brain regions (Table 2. Spheres with a radius of 7.5 mm were conjoined with the thresholded activation from the localizer to construct the ROIs. Activity during the experimental (person-knowledge) tasks did not factor into the localisation procedure.

**Table 2:** ROI sphere centre coordinates. Peak MNI coordinates for regions active in the localiser experiment which was derived from an independent set of participants (N=42; see text) and ROI sizes in voxels after thresholding.

Region	Hemisphere	X	Y	Z	ROI Size
OFA	Left	−33	−88	−10	49
	Right	30	−91	−10	65
FFA	Left	−39	−46	−22	30
	Right	39	17	23	44
pSTS	Left	−48	−49	14	54
	Right	48	−55	14	71
Precuneus	Medial	3	−52	29	81
IFG	Left	−36	20	26	38
	Right	39	17	23	44
ATL	Left	−60	−7	−19	69
	Right	57	−7	−19	81
Amygdala	Left	−21	−10	−13	62
	Right	21	−7	−16	59
dmPFC	Medial	6	59	23	59
vmPFC	Medial	3	50	−19	66
OFC	Left	33	35	−13	58
	Right	−33	35	−13	27
ATFP	Left	33	−10	−40	39
	Right	−36	−10	−34	24
Angular Gyrus	Left	−48	−67	35	68
	Right	42	−64	35	57

In order to remove the effects of reading names alone, within each region we subtracted the activation magnitude during the control condition (1-back famous name matching task) from the task activation.

### 2.7 Multivariate analyses & RSA models

**Fig. 4:**
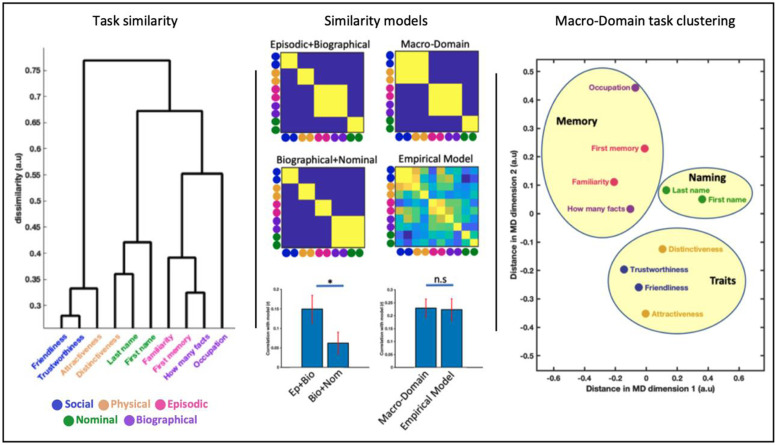
Organisation of person-knowledge. Left: Dendrogram plot reflecting the similarity in network activation patterns. Tasks that are linked with short paths (e.g. those of “trustworthiness” and “friendliness”) signify that patterns across the extended system ROIs during these tasks are similar. Points far apart (e.g. “occupation” and “attractiveness”) mean that these tasks elicit different patterns. Middle: Matrices illustrate hypotheses about cognitive structure expressed as models of expected similarity. Bar graphs show model fit comparisons. Biographical knowledge is represented more like episodic knowledge, than nominal knowledge. The theoretical macro-domain model explains as much variance as the empirical model derived from the face viewing experiment. Right: Multidimensional task similarity structure visualised. MDS plot of task similarity with macro-domain clusters overlaid. Ten cognitive tasks form three groups of cognitions: social and physical trait judgements, tasks involving retrieval of episodic or biographical knowledge, and tasks involving knowledge about names.

**Fig. 5:**
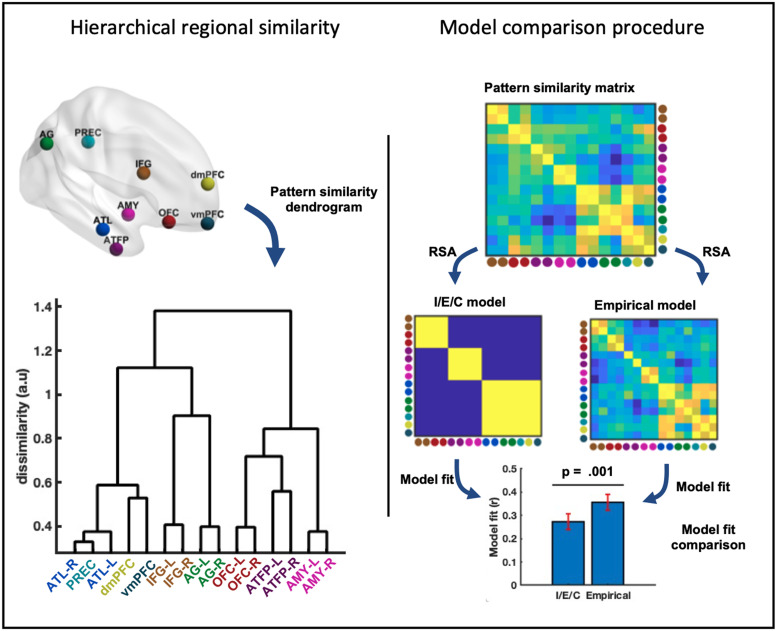
Regional taxonomy of the extended system. Left: Dendrogram showing the clustering of cognitive tuning profiles. First major division in the dendrogram separates internalised cognition regions and IFG from ventromedial regions and OFC. Right: Competing models of regional structure. Average regional similarity during face-cued person-knowledge access (“empirical model”) better explains the data than the theoretical model of the tripartite organisation into internalised, externalised and control regions (“I/E/C” model).

Regional responses were averaged across voxels within each ROI, separately for each task. Average ROI responses to each task were pairwise correlated to obtain a ROI dissimilarity matrix (1-r), which was then entered into Ward hierarchical agglomerative clustering (Ward, 1963). For task similarity analysis the ROI response matrix was transposed before correlating such that the dissimilarity matrix consisted of task dissimilarity across ROIs (Fig. 1b). Importantly - representational similarity (task similarity and region similarity) were calculated for each subject individually and then averaged across subjects only during clustering analyses (rather than averaging the data across subjects and calculating the representational similarity based on these averaged data). We chose this approach as it allowed estimates of variability and error, and allowed inference (Figs. 4 and 5, barplots). The final clustering was calculated based on these averaged RSA spaces (Figs. 4 and 5 dendrograms).

To build models of task and ROI similarity, an almost identical experiment was performed with an independent group of subjects (*N* = 20) using faces instead of names as stimuli (“face experiment” Aglinskas and Fairhall 2020).

In both the current and face experiments participants recalled ten types of knowledge spanning 5 macro-domains (see ”Task” above) prompted by the identities of 40 widely recognizable (famous) individuals. The task macro-domains and identity of stimulus people was held constant across experiments together with other experimental variables (stimulus on screen time, ISI, block duration, etc.). The only differences were (1) Stimulus modality which was either pictures of faces or written names and (2) One of the nominal tasks differed across experiments. Task “How well can you recall this person's full name?” in the face experiment was changed to “How common is this person's last name” for the name experiment.

Task and ROI similarity matrices from the face experiment (Fig. 4, Fig. 5 in Aglinskas and Fairhall 2020) were used as empirical RSA models (in addition to theoretically grounded models, Figs. 4 and 5 “models”), and compared with their respective similarity structures in the current experiment, using a standard RSA model comparison framework (Kriegeskorte et al., 2008). Specifically for each subject we compared the observed similarity (either task or region similarity) with expected (model) similarity, resulting in one r value per subject. We used t-tests to establish significance (one-sample t-test against 0) or to compare between models (paired t-test). We additionally report the r value between the average similarity (task or ROI) and each model tested.

## 3 Results

### 3.1 Behavioural results

Average reaction time (RT) across all tasks was M = 830 ms, SD = 288 ms. Participants responded the fastest during attractiveness judgements (M = 785msec  SD = 116msec), and slowest during “last name” task (883msec SD = 147msec). Reaction times differed across ten tasks F(9153) = 2.52, p = 0.022, but critically, were balanced across task domains F(4,68) = 1.59, p = 0.186. To verify participants’ knowledge about famous people used as stimuli, we analysed their in-scanner behavioural responses during “familiarity” and “occupation” tasks (Table 1). When asked about familiarity, M = 88% SD = 13% of participants indicated at least some degree of familiarity with the stimulus person (between 3 - “little” to 1 - “very much”). Participants correctly identified the occupation of M = 79.4%, SD = 15.6% of people, indicating high levels of knowledge about the people presented. This is consistent with high recall results from the previous study (“face experiment”) where, when presented with faces instead of names, participants reported some degree of familiarity with 87% of stimuli and correctly identified the occupation of M=84% of people presented. Average familiarity ratings were consistent across famous individuals, with no individual deviating more than 2 SD from the mean. Comparing response patterns across conditions that were identical between face and name experiments (all tasks except the nominal) average responses (across participants) were consistent, Spearman's rank correlation r =0.88.

### 3.2 Overall response to name-cued person-knowledge

To ensure that accessing person-knowledge during word reading produced activations broadly consistent with face-cued person-knowledge access - we performed a whole brain analysis, collapsing across task domains. The contrast of all person-knowledge tasks > 1-back famous name matching (voxel threshold p < 0.001, cluster threshold p < 0.05, family-wise error corrected) revealed an expected cluster of activations, broadly consistent with the extended system for face perception (Fig. 2, Table 3). These regions include extended system regions associated with intrinsic cognition: Precuneus (PREC), Angular Gyri (AG), dorsomedial (dmPFC) and ventromedial prefrontal cortices (vmPFC) and left anterior temporal lobe (ATL). Name-cued person-knowledge tasks also recruited a broad cluster of activations over the left prefrontal cortex including the inferior frontal gyrus (IFG) and lateral orbitofrontal cortex (OFC).

**Table 3:** Whole brain activations during semantic access (all person-knowledge tasks > 1-back name matching). For activation clusters larger than 50 voxels, we additionally report individual peaks spaced more than 30 mm apart.

Region	Hemisphere	Cluster statistics			Peak statistics			
		p (FWE)	extent (voxels)	t	p (FWE)	x, y, z (mm, MNI)		
dmPFC	Medial	< 0.001	3076	9.60	< 0.001	−12	32	53
IFG	Left			6.98	< 0.001	−51	26	11
OFC	Left			6.16	< 0.001	−39	17	44
PREC	Medial	< 0.001	541	9.70	< 0.001	0	−49	26
vmPFC	Medial	0.041	56	5.51	0.003	0	50	−19
ATL	Left	< 0.001	134	6.00	< 0.001	−60	−10	−19
AG	Left	< 0.001	301	7.65	< 0.001	−45	−61	26
	Right	< 0.001	266	6.93	< 0.001	51	−67	35
Precentral g.	Left	< 0.001	203	5.44	0.005	−36	−19	53
Cerebellum	Left	0.012	74	5.59	0.002	−36	−76	−40
	Right	< 0.001	441	7.46	< 0.001	36	−79	−40

To further investigate person-knowledge representation in these regions, we extracted response magnitudes for each task, from face-selective ROIs that we defined using an independent dataset (see methods). To assess the overall univariate response during access to person-knowledge (relative to the name matching task), we averaged across all domains of person-knowledge. One-sample t-tests were performed across face-selective ROIs to assess whether each ROI also responded to name stimuli when person-knowledge is accessed (Bonf. adjusted threshold p = .0033). Medial regions (precuneus, dmPFC, vmPFC) were strongly engaged by person-knowledge demands (all t > 3.59, p < .003), compared to a 1-back matching task.  While AG and OFC responded strongly to person-knowledge demands bi-laterally (all t > 3.74, p < .002) - IFG & ATL only showed significant response in the left hemisphere (left hemisphere (t > 4.79, p < .001); right hemisphere (t < 0.86, p > .36). Anterior temporal face patch (ATFP) regions and the amygdalae were, on average, not responsive to person-knowledge demands (t < 1.92, p > .067; but see next section: cognitive tuning). While ANOVA indicated weak left lateralised effects (main effect of hemisphere: F(1,23)=5.94, p=.023) differences in lateralization across regions did not reach significance (hemisphere by region interaction F(5115)=1.43, p=.218).

These results confirm that some regions of the extended system are recruited during name-cued person-knowledge retrieval. However they do not inform about the potentially different role each region plays in accessing knowledge of different domains (e.g. social knowledge, biographical knowledge etc.). To better understand regional contributions to ensemble function - we now look into differential regional activations across diverse person-knowledge tasks.

#### 3.2.1 Regional cognitive tuning

While some regions are not active compared to the name-matching task when all cognitive domains are averaged, it is possible that the pattern of recruitment across domains of person-knowledge contains information about the cognitive profile of these regions. Next we perform a preliminary evaluation of whether the response in these regions varied across the five cognitive domains.

To visualise the graded importance of different cognitive domains within a particular region - we scale each region's domain-specific response, by the total (summed) response across all five domains. While ANOVA in bilateral ROIs revealed a significant 3-way interaction (domain by region by hemisphere f(20,169.6)=2.92, p=.006; Greenhouse-Geisser corrected), for simplicity results are presented collapsed across hemispheres. The variations in regional sensitivity to the different cognitive domains is evident in Fig. 3. To focus on the differential role of network regions in the five knowledge-domains, Fig. 3 depicts the contribution of each knowledge domain above the 1-back name matching control task. It should be noted that it is possible that some information about individuals may be spontaneously retrieved during the name-matching baseline condition and thus reported results may therefore underestimate the overall effect of information access. Some regions, like the precuneus, respond strongly to all domains (all t > 6.88, all p < .001), while others respond weakly to only some domains (e,g, ATFP). Overall, regions exhibit graded patterns of preference, with most regions being involved in most cognitive domains, and no brain regions being driven by a single cognitive domain. However, subtle patterns can be seen in the variations of loadings of particular domains in different brain regions. Next, we exploit these graded patterns with NetRSA to build up both cognitive tuning and regional coordination taxonomies.

### 3.3 Stable cognitive tunings in extended, but not core regions

We next tested whether regional cognitive tunings are consistent during name-cued and face-cued access to person-knowledge. For each region, we used the pattern of activity evoked by the ten person-knowledge tasks during face-cued access (averaged across subjects) as a model, and correlated it to the corresponding regional patterns during name-cued person-knowledge access (for each person individually). Cognitive tasks produce reliably similar patterns during face- and name- cued person-knowledge in the extended system regions (r=0.68, t(23) = 6.22,p < .001). However, when we repeated this process in core regions, where there was no similarity between patterns during face viewing and name reading r=0.20 t(23) = 0.77,p = .448.

### 3.4 Cognitive taxonomy of person-knowledge

We begin with a description of task clustering and its similarities and differences compared to the face-experiment, followed by formal inference in the following section (c.f. “Three macro-domains of person-knowledge”). A wide range of information can be accessed about people. Knowing which tasks are represented similarly can inform the cognitive structure of person-knowledge. The similarity between these diverse cognitive processes in the human brain can be quantified by the similarities in how they recruit different elements of the extended system. Here we used NetRSA to address the systems-level relationship between the ten tasks probed in this study.

At the most basic level (Fig. 4, bottom of the dendrogram tree) - tasks can be seen to group into task-pairs; tasks sampled from the same person knowledge domain (social, physical, episodic, biographical and nominal) produce similar patterns of regions activations and are grouped close together. Higher order clustering (further up the dendrogram tree) reveals relationships not only between tasks, but between task domains. Specifically, biographical and episodic knowledge are more similar to each other than to other tasks. Nominal knowledge forms a distinct cluster - separate from physical and social knowledge tasks, which are grouped together. One exception to this is the distinctiveness task, which clustered tightly with other socio-perceptual judgements in face study, but here seems more closely related to nominal tasks. Multidimensional Scaling (MDS) procedure offers an alternate (two-dimensional) view of task similarity and allows to disambiguate these results. It can be seen in the MDS that the distinctiveness task still shares a substantial amount of pattern similarity with attractiveness and social tasks and is positioned roughly equidistant between nominal and socio-perceptual task clusters. These purely descriptive results are followed by inferential analyses below.

### 3.5 Three macro-domains of person-knowledge

Previously we showed that the cortical representation of biographic knowledge was more shared with episodic than nominal knowledge, a grouping that is also apparent for the name data. To formally assess whether this generalises to name stimuli, we contrast models in which biographical knowledge is grouped with episodic, against one in which the former is grouped with nominal. Results show that biographical knowledge is represented more similarly to episodic knowledge (r=0.40) than nominal knowledge (r=0.19), t(23) = 2.89, p = .008.

During face viewing, we observed that cognitive tasks cluster into three macro-domains: socio-perceptual judgements, episodic and biographical knowledge, and nominal knowledge. We explicitly modelled this task grouping [r=0.64, t(23) = 6.47, p < .001] and see that it captures as much variance as the average face data model itself, the difference between the macro-domain model and the empirical model was not significant, t(23) = 0.18,p = .858. This indicates that the similarity between face and name cued cognitive representation in the extended system can be equivalently captured by this theoretical model. To assess the possible impact of systematic reaction time differences on the observed patterns of neural similarity, we correlated each individual's reaction time similarity and neural task similarity. Results indicate no evidence for a relationship t(17) = −1.24, p=.234, suggesting that RT related effects like time-on-task do not underlie the reported netRSA results.

## 4 Regional taxonomy

### 4.1 Interregional coupling across cognitive domains

To better understand how coordinated regional activity encodes diverse kinds of person-knowledge, we look at patterns of similarity in cognitive tuning profiles across regions – identifying groups of regions responding similarly across tasks. We visualise regional similarity structure by computing a dendrogram, and fit competing RSA models of regional similarity to test hypotheses about network organisation.

Descriptively, hierarchical clustering (dendrogram, Fig. 5) indicates that, consistent with face-cued access to person information, regions associated with internalised cognitive processes (ATL, precuneus, vmPFC and dmPFC) continue to cluster together, as do ventromedial regions (ATFP, amygdala). The OFC and IFG, which clustered with perceptual regions during face-cued access to person information, exhibit a different regional clustering pattern during name-cued access. Here, the IFG loosely clusters with the AG. During face cued information access, AG was instead relatively loosely linked to intrinsic regions. In contrast, the OFC now clusters with ventromedial ATFP and amygdala. The change in coordination profiles of IFG and OFC holds true regardless of whether core system ROIs are included in the clustering analysis (Fig. S3). We next turn to inferential RSA analyses.

Despite these descriptive changes in clustering, regional coordination in the extended systems remains largely similar between names and faces (consider name-cued pattern-similarity and the face-data model; Fig. 5). To formally test the degree of convergence across these two modalities, we compared competing models of regional organisation to the observed data. The face-data model captured significant amounts of variance during name reading t(23) = 10.30, p < .001. While the theoretical model, designed to model the broad tripartite organisation into internalised, externalised and control regions (“I/E/C” model) of the extended system seen during face-cued access to person-knowledge, correlated with observed data (t(23) = 8.24, p < .001), unlike cognitive taxonomy, the theoretical model did not match the empirical model derived from the face data, with the latter capturing significantly more variance (t(23) = 3.75,p = .001). This indicates that subtle information beyond these coarse-scale groupings is also shared between words and faces.

## 5 Discussion

This study investigated the similarities and differences between cortical substrates underlying access to different variants of person-knowledge when cued by words. We used an RSA approach applied across a network of regions (NetRSA) to characterise the inter-regional functional coordination across access to varied forms of person-knowledge. Within an independently localised set of person-selective ROIs, we contrasted these results to a previous study in which access to person-knowledge was cued by faces (Aglinskas and Fairhall, 2020) and observe that: 1) Name-cued access to person-knowledge recruits regions consistent with the face-cued extended system, and that regardless of stimulus modality, these regions are involved in most-all tasks and show relative rather than absolute activation differences across person-knowledge domains. 2) Extended-system regions form groups of functionally similar units, some having stable functional roles across modalities (internalised cognition and ventromedial regions) while some show flexible coupling depending on stimulus demands (IFG and OFC). 3) Consistent with face-cued access, the pattern of regional coordination across tasks groups cognitive domains into three macro-domains: memory, naming and socio-perceptual judgements.

### 5.1 Regional taxonomy

First, we examined the traditional univariate response for access to person-knowledge (relative to 1-back name matching) in the absence of face stimuli. Access to person knowledge elicited activations in key components of the default mode network: medial PFC, the precuneus and bilateral angular gyri. The left lateral ATL, left IFG and bilateral OFC also showed robust activation during name-cued access to person-knowledge. In contrast, in the right IFG, right ATL and bilateral AMY and ATFP, name cued responses were weak or absent, which on the surface implies that these regions do not play a role in access to person-knowledge in the absence of faces.

However, despite absences of overall increase in activation in some regions, subtle yet systematic variations in response profiles were evident. These variations were used for the multivariate NetRSA approach to quantify the relationship between the response profiles of elements of the extended system. Qualitatively, we see that, for all regions, left and right homologues cluster together into functional units. This pattern persists in cases where the overall effect in the left hemisphere region is significant and in the right hemisphere region it is not (IFG, ATL), or when neither regions show a significant overall increase in response relative to 1-back name matching (ATFP). This demonstrates that the varied forms of person-knowledge drive these regions in a consistent way and indicates that they might contribute to the retrieval of knowledge about conspecifics even in the absence of an overall response. Importantly, qualitative clustering results are solely descriptive, limiting the conclusions that can be drawn from it. We quantitatively tested select hypotheses using inferential RSA statistics (Figs. 4 and 5).

As can be seen in Fig. 3, regional response patterns across cognitive domains show that most regions are involved in most person-knowledge domains, that no regions are responsive to a particular domain, and that there are subtle variations between cognitive tuning across regions. These subtle variations accumulate into hierarchical clusters of inter-regional coordination across tasks: intrinsic, ventromedial and a loose grouping comprising the IFG and angular gyri.

The intrinsic subcomponent of the extended system is highly conserved across face- and name-cued access to person-knowledge. This is consistent with the location of these regions in DMN. This set of brain regions is involved in internalised processes that are largely independent of interactions with the outside world (Spreng and Grady, 2010) and are therefore expected to be relatively insensitive to the modality of the simulus. One exception to this pattern was the AG. This region was loosely grouped with the other elements of the intrinsic system during face-cued access to person-knowledge (Aglinskas and Fairhall, 2020). In the present study, we see that this grouping is lost, with the region instead loosely grouping with the IFG. The IFG responds selectively to faces when identity must be extracted from a face but not when a purely perceptual judgement is made, suggesting that this region is involved in identity processing or identity extraction (Ubaldi and Fairhall, 2021a). During face-cued access to person-knowledge, the functional profile of the IFG was similar to the functional profiles of core regions, which we attributed to top-down control of perceptual processes (Aglinskas and Fairhall, 2020), as supported by studies showing that TMS stimulation to IFG modulates the response in core regions (Renzi et al., 2013) and findings of common temporal tuning profiles between IFG and core regions (Bortolon et al., 2015; Ubaldi and Fairhall, 2020). In the present study, where top-down perceptual face-processing demands are absent, more subtle, non-perceptual aspects and coordinations across tasks may be more apparent, and the relationship between IFG and other regions may become more evident. The reason this leads to the loose coupling between IFG and AG observed here is uncertain. While these regions are commonly active in semantic processes (Binder and Desai, 2011), and AG and dorsolateral prefrontal regions including IFG are associated with processes like memory post-retrieval monitoring (Achim and Lepage, 2005), as well as attentional orienting (Corbetta and Shulman, 2002). The reason for their common coactivation patterns in the present context is uncertain. Like the IFG, the OFC grouped with core perceptual systems during face-cued access. Here, in the absence of these perceptual demands, OFC groups with ventromedial regions, the ATFP and amygdala. This grouping may reflect the role of the OFC in reward processes (Adolphs, 2002; Bortolon et al., 2015) and it's connectivity with limbic systems, such as the amygdala (Baxter and Murray, 2002; Dixon et al., 2017).

An open question is the extent to which reading names might automatically trigger access to face information and face imagery, potentially driving similar effects under name-reading and face-viewing conditions. Such an assertion gains support from findings that familiar speakers can drive effects in ventral face-selective regions (von Kriegstein et al., 2008; Schall et al., 2013; although see also Fairhall et al. 2014, Wang et al. 2016, Bi et al. 2016). The finding of an absence of similarity between patterns during face viewing and name reading in the core system (OFA, FFA & pSTS), where one would expect face information to be most strongly represented, argues against this role of imagery type processes.

The focus of this work was on the preservation of overall regional organisation across face- and name-cued access to person-knowledge. Despite the alterations in clustering of specific regions (IFG and OFC), the theoretic model of regional groupings that best fit the face-cued access data (internalised/externalised/control) (Aglinskas and Fairhall, 2020), continued to robustly capture regional groupings in the name-cued data. At the same time, there was additional information present in the empirical model, suggesting that the commonality between face- and name-cued access to person-knowledge is encoded in finer-grained patterns than this coarse three-cluster model. This means that person-knowledge, as it is shared across face and name cued processing, is best captured in the subtle variations of interregional activity rather than broad functional subdivisions of this network. This, in turn, suggests that person-knowledge does not resolve into “modular” subnetworks - but instead additionally relies on subtle variations in the coordinated activity within and between these subnetworks.

### 5.2 Cognitive taxonomy

When we think about a person there is a range of different knowledge we can access. NetRSA allows us to probe the commonalities and differences in how these forms of knowledge are accomplished within the brain. We observed the relationship between these cognitive domains across the extended system to be highly conserved across name and face cued semantic access. As is the case in face-cued semantic access, cognitive domains were seen to group in memory related processes (semantic, episodic), trait based processes (social, physical) and nominal knowledge. These macro-domains fall broadly within hypothesised domain-specificity boundaries (Spunt and Adolphs, 2017), suggesting that declarative memory (episodic, semantic tasks) and language (nominal tasks) are part of the “cognitive”macro-domain, while facial visual knowledge (physical tasks) and theory of mind (social tasks) are part of the “social” macro-domain. The observation that the macro-domain model explains as much variance as the empirical model shows that differences in the neural representation of these tasks in the extended system can be fully explained by a discrete model without a loss of accuracy. Thus, unlike the pattern of regional interactions, the relationship between cognitive processes is both conserved across name and face cued access and is equivalently captured by coarse-grained theoretical divisions.

Semantic knowledge incorporates both factual world knowledge and the meaning contained in words (Tulving, 1972). This prompted us to test whether biographical semantic knowledge relied on neural substrates more similar to those associated with episodic knowledge or with nominal knowledge. Replicating results seen in the face-cued access to person knowledge (Aglinskas and Fairhall, 2020), the link between episodic and semantic forms of memory was seen to be stronger than the link between semantic knowledge and word meaning, suggesting that this form of semantic access is more closely related to other forms of memory than systems that link words to individuals.

The observed cognitive taxonomy informs the relationships between specific cognitive domains. Social judgements are more similar to perceptual ones than to memory or name related judgements. There is evidence linking social and perceptual processes when related to faces. Attractive people are rated as more trustworthy (Halo effect; Nisbett and Wilson, 1977; Wetzel et al., 1981) and parametrically moduled physical facial traits similarly modulate judgement of social traits (dominance, trustworthiness; Oosterhof and Todorov 2008). In this context, the grouping of social and perceptual cognitive processes in face-cued access is unsurprising. However, the preservation of this grouping in name-cued stimuli (which do not involve face perception) indicates the coupling of these processes that extends beyond the perceptual, potentially reflecting the intrinsic bias in the allocation of social characteristics based on appearance (Wilson and Eckel, 2006).

While the cognitive taxonomies are by-and-large consistent across name and face stimuli, the exception that occurred in the physical knowledge-domain can inform us about flexibility in representations across input modalities. In the present study, attractiveness was seen to cluster with the social traits (trustworthiness and friendliness). However, it no longer clustered with the other physical task cohort ‘distinctiveness’, as was the case when face stimuli cued knowledge access. This disparity suggests that when face stimuli are present, physicality judgments are made in more similar way relying on common neural substrates, while when the physical face stimuli is absent, knowledge access and representation rely more heavily on differing neural mechanisms (such as the social domain in the case of attractiveness judgements). While the work considers multiple domains of knowledge related to other people, interpretations are constrained to this context. Future work is needed to understand representation of the diverse aspects of information processing, such as action intent and meaning, semantic processes related to understanding incomplete incoming information or when content spans multiple modalities.

Collectively, these results illustrate the capacity of NetRSA to consistently reveal the relationship between different cognitive processes based upon their representations across brain networks. The construction of such cognitive taxonomies is a challenge for cognitive neuroscience. While the similarity between conceptual or semantic processes can, in part, be captured by empirical or computational models (e.g. word embeddings, alexnet, Krizhevsky et al. 2012, Mikolov et al. 2013), diverse cognitive processes are more challenging to quantify. Traditionally, dissimilarities between different cognitive processes have to be derived from theory (such as Baddeley and Hitch 1974, Spunt and Adolphs 2017). NetRSA provides a highly flexible alternative to uncover the taxonomic structure of different cognitive processes.

## 6 Conclusion

The current study addressed the way person-knowledge is represented in the extended system for face-perception. We show that common elements are recruited for both face- and name- cued access to person-knowledge. Multivariate, network-level analysis (NetRSA) revealed that cortical representation of different person-knowledge domains is largely preserved within the extended system regardless of stimulus demands. Diverse types of person-knowledge form three macro-domains sharing common cortical signatures: socio-perceptual information (social and perceptual judgements), memory-related processes (biographical and semantic knowledge) and nominal knowledge. The stability of this cognitive taxonomy is facilitated by the flexible organization of the extended system. Some elements (IFG, OFC) change their coordination patterns, likely to enable the access of stored conceptual content cued by markedly diverse perceptual inputs (faces or names). Conversely - regions associated with internalised cognitive processes were largely agnostic about the modality of presentation. Taken together, these results suggest that the diverse repertoire of person-knowledge, and the ability to access stored information across heterogenous prompts (seeing faces or reading names) - is enabled by the coordinated activity of the extended system regions, rather than relying on any individual region.

## Data and code availability statement

Binarized ROI masque, domain-specific t-statistic maps and ROI data are freely available on the Open Science Framework (OSF) at the following URL:https://osf.io/ph349/?view_only=df5ded5ea9e84929996aa1644d3e77c9.

## CRediT authorship contribution statement

**Aidas Aglinskas:** Formal analysis, Writing – original draft, Investigation. **Scott L. Fairhall:** Conceptualization, Formal analysis, Writing – original draft, Funding acquisition, Supervision.

## Data Availability

Data are available on the Open Science Framework (OSF) at the following URL: https://osf.io/ph349/?view_only=df5ded5ea9e84929996aa1644d3e77c9.

## Appendix Supplementary materials

Image, application 1
