# Supplementary material for: Similar representation of names and faces in the network for person perception
Source: Neuroimage. 2023 Jul 1;274:120100. doi: 10.1016/j.neuroimage.2023.120100 (PMC10199409; doi:10.1016/j.neuroimage.2023.120100)
Supplement: Supplementary file 1 [file mmc1.pdf]

Data and code availability statement

Binarized ROI mask, domain-specific t-statistic maps and ROI data are freely available on the Open Science Framework (OSF) at the following URL:
https://osf.io/ph349/?view_only=df5ded5ea9e84929996aa1644d3e77c9
